# Poly[[aqua­tris­(μ_4_-benzene-1,2-dicarboxyl­ato)dilanthanum(III)] hemihydrate]

**DOI:** 10.1107/S1600536811036282

**Published:** 2011-09-14

**Authors:** Shie Fu Lush, Fwu Ming Shen

**Affiliations:** a Department of General Education Center, Yuanpei University, HsinChu, 30015 Taiwan; b Department of Biotechnology, Yuanpei University, No. 306, Yuanpei St, HsinChu, 30015 Taiwan

## Abstract

The asymmetric unit of the title coordination polymer, {[La_2_(C_8_H_4_O_4_)_3_(H_2_O)]·0.5H_2_O}_*n*_, contains two independent La^III^ atoms, one of which is surrounded by eight carboxyl­ate-O atoms from six benzene-1,2-dicarboxyl­ate (BDC) anions in a bicapped trigonal–prismatic geometry. The other La^III^ atom is nine-coordinated in a tricapped trigonal–prismatic geometry, formed by eight carboxyl­ate-O atoms from six BDC anions and a coordinated water mol­ecule. The BDC anions bridge the La^III^ atoms, forming a two-dimensional polymeric complex parallel to (001). The crystal structure contains weak O—H⋯O and non-classical C—H⋯O hydrogen bonds. A C—H⋯π inter­action is also present in the crystal structure. The uncoordinated water molecule shows half-occupation.

## Related literature

For a related structure, see: Wang *et al.* (2009 ▶).
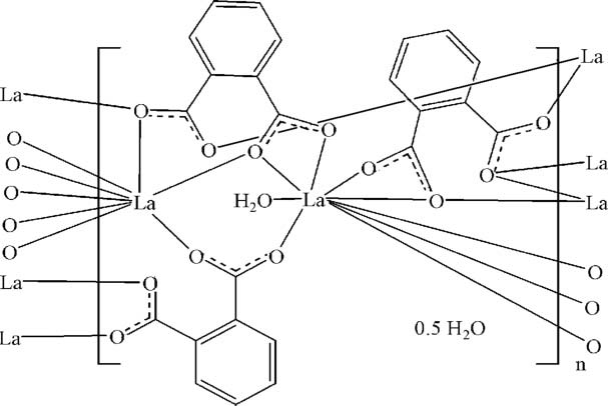


## Experimental

### 

#### Crystal data

[La_2_(C_8_H_4_O_4_)_3_(H_2_O)]·0.5H_2_O
*M* *_r_* = 1594.36
Triclinic, 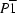

*a* = 8.6269 (19) Å
*b* = 10.5832 (17) Å
*c* = 14.323 (2) Å
α = 97.271 (18)°
β = 102.199 (6)°
γ = 104.489 (8)°
*V* = 1215.1 (4) Å^3^
*Z* = 1
Mo *K*α radiation
μ = 3.54 mm^−1^
*T* = 150 K
0.47 × 0.24 × 0.04 mm

#### Data collection

Nonius KappaCCD diffractometer
Absorption correction: multi-scan (*SCALEPACK*; Otwinowski & Minor, 1997 ▶) *T*_min_ = 0.287, *T*_max_ = 0.871
8765 measured reflections
4145 independent reflections
3430 reflections with *I* > 2σ(*I*)
*R*_int_ = 0.032

#### Refinement

*R*[*F* ^2^ > 2σ(*F* ^2^)] = 0.037
*wR*(*F* ^2^) = 0.087
*S* = 1.11
4145 reflections
356 parameters
12 restraints
H-atom parameters constrained
Δρ_max_ = 1.25 e Å^−3^
Δρ_min_ = −0.91 e Å^−3^

### 

Data collection: *COLLECT* (Nonius, 2000 ▶); cell refinement: *SCALEPACK* (Otwinowski & Minor, 1997 ▶); data reduction: *DENZO* (Otwinowski & Minor, 1997 ▶) and *SCALEPACK*; program(s) used to solve structure: *SHELXS97* (Sheldrick, 2008 ▶); program(s) used to refine structure: *SHELXL97* (Sheldrick, 2008 ▶); molecular graphics: *PLATON* (Spek, 2009 ▶); software used to prepare material for publication: *PLATON*.

## Supplementary Material

Crystal structure: contains datablock(s) global, I. DOI: 10.1107/S1600536811036282/xu5316sup1.cif

Structure factors: contains datablock(s) I. DOI: 10.1107/S1600536811036282/xu5316Isup2.hkl

Additional supplementary materials:  crystallographic information; 3D view; checkCIF report

## Figures and Tables

**Table 1:** Selected bond lengths (Å)

La1—O1	2.599 (4)
La1—O2	2.645 (5)
La1—O3	2.695 (6)
La1—O4	2.613 (4)
La1—O5^i^	2.617 (4)
La1—O7^ii^	2.439 (5)
La1—O17^iii^	2.543 (4)
La1—O20	2.478 (5)
La1—O22	2.611 (4)
La2—O1	2.535 (4)
La2—O4^iv^	2.482 (4)
La2—O5^iv^	2.625 (4)
La2—O6^iii^	2.466 (4)
La2—O8^v^	2.549 (5)
La2—O17	2.608 (4)
La2—O18^iii^	2.495 (4)
La2—O19	2.417 (5)

Symmetry codes: (i) 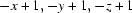; (ii) 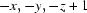; (iii) 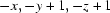; (iv) 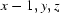; (v) 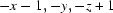.

**Table 2:** Hydrogen-bond geometry (Å, °) *Cg* is the centroid of the C2–C7 ring.

*D*—H⋯*A*	*D*—H	H⋯*A*	*D*⋯*A*	*D*—H⋯*A*
O22—H22*A*⋯O1^ii^	0.90	2.20	3.001 (6)	148
O22—H22*B*⋯O20^ii^	0.88	2.15	3.009 (8)	164
O27—H27*A*⋯O2^vi^	0.91	2.38	3.17 (2)	146
O27—H27*B*⋯O3^i^	0.88	1.86	2.69 (2)	158
C3—H3⋯O27^vii^	0.93	2.15	2.96 (2)	145
C16—H16⋯O2^i^	0.93	2.58	3.331 (10)	138
C19—H19⋯*Cg*^v^	0.93	2.98	3.902 (9)	169

Symmetry codes: (i) 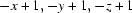; (ii) 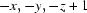; (v) 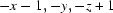; (vi) 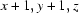; (vii) 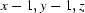.

**Fig. 1.**
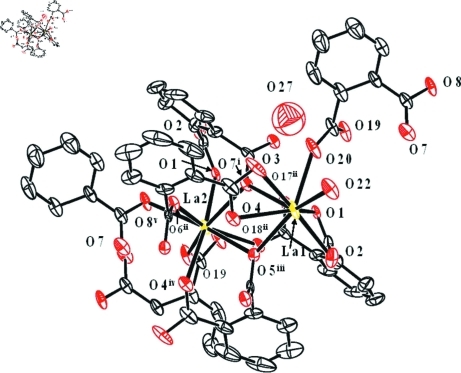
View of the title compound with the atom numbering scheme. Displacement ellipsoids for non-H atoms are drawn at the 50% probability level. H atoms have been omitted for clarity. Symmetry codes:(i) -x, -y, -z + 1; (ii) -x, -y + 1, -z + 1; (iii) -x + 1, -y + 1, -z + 1; (iv) x - 1, y, z; (v) -x - 1, -y, -z + 1.

**Fig. 2.**
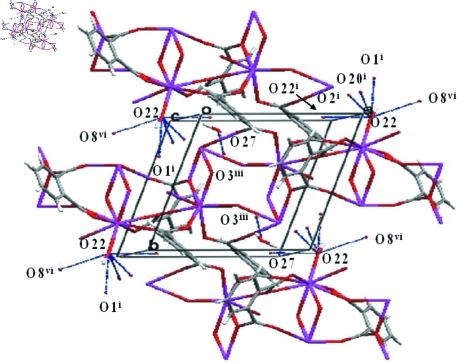
The molecular packing for the title compound, viewed along the c axis. Hydrogen bonds are shown as dashed lines.

## supplementary crystallographic information

### Comment

Benzene-1,2-dicarboxylic acid (H_2_BDC) are widely used in the construction of coordination polymers due to their capability of acting as bridging ligands in various coordination modes. But to the best of our knowledge, H_2_BDC is seldom involved in lanthanide complexes (Wang *et al.*, 2009). In this paper, we describe the hydrothermal synthesis and structure properties of a lanthanide phthalate coordination complex {[La_2_(C_8_H_4_O_4_)_3_(H_2_O)].0.5 H_2_O}_n_.

The molecular structure of the title compound is shown in Fig. 1.There are two independent lanthanum ions in the asymmetric unit. The La(1) ion is nine-coordinated with O_9_ donors sets to form tricapped trigonal prismatic geometries by eight carboxylate O atoms and one water molecule, where La(2) ion is eight-coordinated with O_8_ donors sets to form distorted bicapped trigonal-prismatic geometries by eight carboxylate O atoms, from six benzene-1,2-dicarboxylate anions. The selected bond lengths (Å) of title compound are listed in Table 1. The two La^III^ cations are separated by a non-bonding distance of 4.453 (9) and 4.419 (10) Å. The benzene-1,2-dicarboxylate anions bridge the La^III^ cations, forming a two-dimensional polymeric complex.

There are extensive intermolecular O—H···O and weak C—H···O hydrogen bonds, which cause the stability of the crystal structure (Fig. 2, Table 2). There are no π-π stacking interactions in the title compound. Furthermore, there is C—H···π interaction between C—H group of the BDC ligand, with an C—H···centroid distance of 3.902 (9) Å [C19—H19···*Cg*1^v^(C2—C7)] (Symmetry code:-1-*X*, –Y, 1-*Z*).

### Experimental

LaCl_3_.6H_2_O (0.0868 g, 0.20 mmol), benzene-1,2-dicarboxylic acid (0.0348 g, 0.20 mmol) and 1,2-bis(4-pyridyl)ethane were mixed in 10 ml of deionized water. After stirring for 30 min, the mixture was placed in a 23 ml Teflon-lined reactor, heated at 453 K for 48 h, then cooled slowly to room temperature. The colorless transparent single crystals of the title compound were obtained in 36.76% yield (based on La).

### Refinement

The site occupancy factor of the lattice water O27 was refined to 0.46 (3), and was set as 0.5 at the final cycles of refinement. Water H atoms were placed in calculated positions and refined in riding mode with *U*_iso_(H)= 1.5*U*_eq_(O). Other H atoms were positioned geometrically with C—H = 0.93 Å and refined using a riding model with *U*_iso_(H) = 1.2*U*_eq_(C).

### Crystal data

**Table:**

[La_2_(C_8_H_4_O_4_)_3_(H_2_O)]·0.5H_2_O	*Z* = 1
*M**_r_* = 1594.36	*F*(000) = 762
Triclinic, *P*1	*D*_x_ = 2.179 Mg m^−^^3^
Hall symbol: -P 1	Mo *K*α radiation, λ = 0.71073 Å
*a* = 8.6269 (19) Å	Cell parameters from 4145 reflections
*b* = 10.5832 (17) Å	θ = 2.0–25.0°
*c* = 14.323 (2) Å	µ = 3.54 mm^−^^1^
α = 97.271 (18)°	*T* = 150 K
β = 102.199 (6)°	Prism, colorless
γ = 104.489 (8)°	0.47 × 0.24 × 0.04 mm
*V* = 1215.1 (4) Å^3^	

### Data collection

**Table:**

Nonius KappaCCD diffractometer	4145 independent reflections
Radiation source: fine-focus sealed tube	3430 reflections with *I* > 2σ(*I*)
graphite	*R*_int_ = 0.032
Detector resolution: 9 pixels mm^-1^	θ_max_ = 25.0°, θ_min_ = 2.0°
ω/2θ scans	*h* = −10→10
Absorption correction: multi-scan (*SCALEPACK*; Otwinowski & Minor, 1997)	*k* = −12→12
*T*_min_ = 0.287, *T*_max_ = 0.871	*l* = −17→16
8765 measured reflections	

### Refinement

**Table:**

Refinement on *F*^2^	Primary atom site location: structure-invariant direct methods
Least-squares matrix: full	Secondary atom site location: difference Fourier map
*R*[*F*^2^ > 2σ(*F*^2^)] = 0.037	Hydrogen site location: inferred from neighbouring sites
*wR*(*F*^2^) = 0.087	H-atom parameters constrained
*S* = 1.11	*w* = 1/[σ^2^(*F*_o_^2^) + (0.0369*P*)^2^ + 2.5018*P*] where *P* = (*F*_o_^2^ + 2*F*_c_^2^)/3
4145 reflections	(Δ/σ)_max_ = 0.002
356 parameters	Δρ_max_ = 1.25 e Å^−^^3^
12 restraints	Δρ_min_ = −0.91 e Å^−^^3^

### Special details

**Table:**

Geometry. Bond distances, angles *etc*. have been calculated using the rounded fractional coordinates. All su's are estimated from the variances of the (full) variance-covariance matrix. The cell e.s.d.'s are taken into account in the estimation of distances, angles and torsion angles
Refinement. Refinement on *F*^2^ for ALL reflections except those flagged by the user for potential systematic errors. Weighted *R*-factors *wR* and all goodnesses of fit *S* are based on *F*^2^, conventional *R*-factors *R* are based on *F*, with *F* set to zero for negative *F*^2^. The observed criterion of *F*^2^ > σ(*F*^2^) is used only for calculating -*R*-factor-obs *etc*. and is not relevant to the choice of reflections for refinement. *R*-factors based on *F*^2^ are statistically about twice as large as those based on *F*, and *R*-factors based on ALL data will be even larger.

### Fractional atomic coordinates and isotropic or equivalent isotropic displacement parameters (Å^2^)

**Table:**

	*x*	*y*	*z*	*U*_iso_*/*U*_eq_	Occ. (<1)
La1	0.16724 (4)	0.22260 (3)	0.50902 (3)	0.0255 (1)	
La2	−0.30351 (4)	0.34775 (3)	0.49673 (3)	0.0181 (1)	
O1	−0.0850 (4)	0.2911 (4)	0.4193 (3)	0.0186 (13)	
O2	0.0101 (5)	0.1690 (4)	0.3225 (4)	0.0377 (16)	
O3	0.3563 (6)	0.2580 (6)	0.6913 (4)	0.048 (2)	
O4	0.4799 (5)	0.3465 (4)	0.5867 (3)	0.0239 (14)	
O5	0.7181 (5)	0.5887 (4)	0.5813 (3)	0.0214 (14)	
O6	0.5239 (5)	0.6448 (4)	0.6419 (3)	0.0233 (14)	
O7	−0.3449 (6)	−0.1149 (4)	0.5627 (4)	0.0351 (18)	
O8	−0.5707 (5)	−0.1219 (4)	0.6165 (4)	0.0312 (16)	
O17	−0.1888 (5)	0.5423 (4)	0.4122 (3)	0.0208 (14)	
O18	0.0471 (5)	0.5575 (4)	0.3684 (3)	0.0217 (14)	
O19	−0.2597 (5)	0.1804 (4)	0.5913 (4)	0.0313 (16)	
O20	0.0031 (5)	0.1774 (4)	0.6299 (4)	0.0369 (16)	
O22	0.1603 (6)	−0.0025 (4)	0.5669 (4)	0.0436 (18)	
C1	−0.0786 (7)	0.2446 (6)	0.3327 (6)	0.029 (2)	
C2	−0.1747 (7)	0.2850 (7)	0.2501 (5)	0.032 (2)	
C3	−0.2494 (9)	0.1966 (9)	0.1623 (6)	0.050 (3)	
C4	−0.3483 (10)	0.2326 (13)	0.0877 (6)	0.071 (4)	
C5	−0.3728 (10)	0.3569 (13)	0.1003 (6)	0.067 (4)	
C6	−0.2982 (8)	0.4457 (9)	0.1859 (5)	0.040 (3)	
C7	−0.1968 (7)	0.4135 (7)	0.2618 (5)	0.028 (2)	
C8	−0.1036 (7)	0.5116 (6)	0.3539 (5)	0.0192 (19)	
C9	0.4775 (8)	0.3355 (7)	0.6748 (6)	0.033 (2)	
C10	0.6412 (7)	0.5955 (6)	0.6477 (5)	0.0205 (19)	
C11	0.6971 (8)	0.5475 (8)	0.7397 (5)	0.032 (2)	
C12	0.6165 (8)	0.4227 (8)	0.7530 (5)	0.037 (3)	
C13	0.6653 (10)	0.3832 (12)	0.8412 (7)	0.069 (4)	
C14	0.7926 (11)	0.4676 (16)	0.9153 (7)	0.091 (6)	
C15	0.8710 (11)	0.5890 (14)	0.9012 (7)	0.079 (5)	
C16	0.8232 (9)	0.6330 (9)	0.8153 (6)	0.046 (3)	
C17	−0.4155 (8)	−0.0836 (6)	0.6270 (5)	0.0254 (19)	
C18	−0.3075 (7)	−0.0081 (6)	0.7229 (5)	0.027 (2)	
C19	−0.3358 (9)	−0.0561 (7)	0.8052 (6)	0.040 (3)	
C20	−0.2256 (10)	−0.0039 (9)	0.8933 (7)	0.059 (3)	
C21	−0.0847 (11)	0.1003 (10)	0.9026 (7)	0.070 (4)	
C22	−0.0567 (9)	0.1496 (8)	0.8216 (7)	0.055 (3)	
C23	−0.1669 (7)	0.0972 (6)	0.7317 (6)	0.033 (2)	
C24	−0.1373 (7)	0.1562 (6)	0.6441 (6)	0.028 (2)	
O27	0.717 (3)	0.922 (2)	0.1959 (16)	0.163 (9)*	0.500
H3	−0.23260	0.11290	0.15400	0.0600*	
H4	−0.39840	0.17330	0.02890	0.0860*	
H5	−0.44080	0.38060	0.05010	0.0810*	
H6	−0.31620	0.52910	0.19300	0.0480*	
H13	0.61180	0.29940	0.85010	0.0830*	
H14	0.82430	0.44130	0.97420	0.1090*	
H15	0.95910	0.64440	0.95050	0.0940*	
H16	0.87490	0.71840	0.80830	0.0550*	
H19	−0.43070	−0.12450	0.80030	0.0480*	
H20	−0.24470	−0.03830	0.94780	0.0710*	
H21	−0.01040	0.13620	0.96300	0.0840*	
H22	0.03760	0.21900	0.82730	0.0660*	
H22A	0.17560	−0.07490	0.58980	0.0650*	
H22B	0.10320	−0.04070	0.50710	0.0650*	
H27A	0.82020	0.96320	0.23430	0.2440*	0.500
H27B	0.67000	0.87010	0.23130	0.2440*	0.500

### Atomic displacement parameters (Å^2^)

**Table:**

	*U*^11^	*U*^22^	*U*^33^	*U*^12^	*U*^13^	*U*^23^
La1	0.0128 (2)	0.0071 (2)	0.0613 (3)	0.0054 (1)	0.0149 (2)	0.0089 (2)
La2	0.0126 (2)	0.0076 (2)	0.0386 (3)	0.0058 (1)	0.0113 (2)	0.0063 (2)
O1	0.0111 (19)	0.0089 (19)	0.036 (3)	0.0011 (16)	0.0098 (18)	0.0016 (19)
O2	0.021 (2)	0.024 (2)	0.062 (4)	0.009 (2)	0.008 (2)	−0.015 (2)
O3	0.022 (3)	0.069 (4)	0.069 (4)	0.013 (3)	0.020 (3)	0.053 (3)
O4	0.017 (2)	0.022 (2)	0.042 (3)	0.0137 (18)	0.012 (2)	0.015 (2)
O5	0.017 (2)	0.018 (2)	0.029 (3)	0.0066 (17)	0.0060 (19)	0.0004 (19)
O6	0.017 (2)	0.014 (2)	0.042 (3)	0.0082 (18)	0.010 (2)	0.005 (2)
O7	0.034 (3)	0.017 (2)	0.069 (4)	0.016 (2)	0.030 (3)	0.015 (2)
O8	0.017 (2)	0.010 (2)	0.070 (4)	0.0035 (18)	0.020 (2)	0.005 (2)
O17	0.017 (2)	0.015 (2)	0.037 (3)	0.0098 (17)	0.0123 (19)	0.0093 (19)
O18	0.017 (2)	0.017 (2)	0.036 (3)	0.0084 (18)	0.0109 (19)	0.0081 (19)
O19	0.014 (2)	0.016 (2)	0.066 (4)	0.0068 (18)	0.006 (2)	0.017 (2)
O20	0.019 (2)	0.022 (2)	0.080 (4)	0.0089 (19)	0.020 (2)	0.028 (3)
O22	0.039 (3)	0.017 (2)	0.092 (4)	0.018 (2)	0.033 (3)	0.024 (3)
C1	0.015 (3)	0.016 (3)	0.051 (5)	0.000 (3)	0.012 (3)	−0.010 (3)
C2	0.010 (3)	0.041 (4)	0.037 (5)	0.001 (3)	0.007 (3)	−0.012 (3)
C3	0.023 (4)	0.071 (6)	0.044 (5)	0.003 (4)	0.015 (4)	−0.023 (4)
C4	0.028 (5)	0.129 (10)	0.031 (6)	−0.007 (6)	0.013 (4)	−0.029 (6)
C5	0.026 (4)	0.151 (11)	0.020 (5)	0.013 (6)	0.010 (4)	0.015 (6)
C6	0.022 (4)	0.076 (6)	0.028 (5)	0.017 (4)	0.011 (3)	0.020 (4)
C7	0.015 (3)	0.046 (4)	0.025 (4)	0.012 (3)	0.009 (3)	0.002 (3)
C8	0.019 (3)	0.016 (3)	0.031 (4)	0.009 (3)	0.012 (3)	0.017 (3)
C9	0.018 (3)	0.040 (4)	0.056 (5)	0.020 (3)	0.015 (3)	0.031 (4)
C10	0.008 (3)	0.017 (3)	0.030 (4)	−0.003 (2)	0.003 (3)	−0.003 (3)
C11	0.017 (3)	0.063 (5)	0.026 (4)	0.023 (3)	0.011 (3)	0.008 (4)
C12	0.017 (3)	0.068 (5)	0.037 (5)	0.017 (4)	0.011 (3)	0.029 (4)
C13	0.031 (5)	0.133 (9)	0.067 (7)	0.033 (6)	0.025 (5)	0.060 (7)
C14	0.027 (5)	0.216 (15)	0.044 (6)	0.039 (7)	0.014 (5)	0.059 (8)
C15	0.024 (5)	0.178 (13)	0.033 (6)	0.041 (7)	−0.002 (4)	0.003 (7)
C16	0.023 (4)	0.083 (6)	0.036 (5)	0.026 (4)	0.007 (3)	0.002 (4)
C17	0.024 (3)	0.011 (3)	0.048 (4)	0.008 (2)	0.014 (3)	0.016 (3)
C18	0.017 (3)	0.019 (3)	0.052 (5)	0.008 (3)	0.015 (3)	0.014 (3)
C19	0.027 (4)	0.033 (4)	0.060 (6)	0.002 (3)	0.015 (4)	0.016 (4)
C20	0.047 (5)	0.061 (6)	0.069 (7)	0.005 (4)	0.012 (5)	0.036 (5)
C21	0.045 (5)	0.080 (7)	0.061 (7)	−0.016 (5)	−0.008 (5)	0.031 (6)
C22	0.025 (4)	0.053 (5)	0.076 (7)	−0.006 (4)	0.002 (4)	0.030 (5)
C23	0.016 (3)	0.027 (3)	0.060 (5)	0.010 (3)	0.008 (3)	0.022 (4)
C24	0.016 (3)	0.013 (3)	0.063 (5)	0.008 (3)	0.016 (3)	0.015 (3)

### Geometric parameters (Å, °)

**Table:**

La1—O1	2.599 (4)	C2—C7	1.414 (10)
La1—O2	2.645 (5)	C3—C4	1.377 (13)
La1—O3	2.695 (6)	C4—C5	1.380 (18)
La1—O4	2.613 (4)	C5—C6	1.369 (12)
La1—O5^i^	2.617 (4)	C6—C7	1.380 (10)
La1—O7^ii^	2.439 (5)	C7—C8	1.501 (10)
La1—O17^iii^	2.543 (4)	C9—C12	1.470 (11)
La1—O20	2.478 (5)	C10—C11	1.500 (10)
La1—O22	2.611 (4)	C11—C16	1.392 (11)
La2—O1	2.535 (4)	C11—C12	1.387 (12)
La2—O4^iv^	2.482 (4)	C12—C13	1.395 (13)
La2—O5^iv^	2.625 (4)	C13—C14	1.381 (16)
La2—O6^iii^	2.466 (4)	C14—C15	1.36 (2)
La2—O8^v^	2.549 (5)	C15—C16	1.388 (14)
La2—O17	2.608 (4)	C17—C18	1.484 (10)
La2—O18^iii^	2.495 (4)	C18—C19	1.385 (10)
La2—O19	2.417 (5)	C18—C23	1.397 (9)
O1—C1	1.293 (9)	C19—C20	1.362 (13)
O2—C1	1.253 (8)	C20—C21	1.391 (14)
O3—C9	1.244 (9)	C21—C22	1.373 (14)
O4—C9	1.286 (9)	C22—C23	1.381 (12)
O5—C10	1.274 (8)	C23—C24	1.511 (11)
O6—C10	1.242 (8)	C3—H3	0.9300
O7—C17	1.261 (9)	C4—H4	0.9300
O8—C17	1.268 (9)	C5—H5	0.9300
O17—C8	1.286 (8)	C6—H6	0.9300
O18—C8	1.230 (8)	C13—H13	0.9300
O19—C24	1.265 (9)	C14—H14	0.9300
O20—C24	1.240 (8)	C15—H15	0.9300
O22—H22B	0.8800	C16—H16	0.9300
O22—H22A	0.9000	C19—H19	0.9300
O27—H27B	0.8800	C20—H20	0.9300
O27—H27A	0.9100	C21—H21	0.9300
C1—C2	1.470 (10)	C22—H22	0.9300
C2—C3	1.389 (11)		
			
O1—La1—O2	49.50 (14)	La1^iii^—O17—C8	125.3 (4)
O1—La1—O3	138.66 (15)	La2^iii^—O18—C8	141.3 (4)
O1—La1—O4	134.61 (13)	La2—O19—C24	136.6 (4)
O1—La1—O20	83.93 (14)	La1—O20—C24	145.0 (5)
O1—La1—O22	126.61 (15)	La1—O22—H22B	86.00
O1—La1—O7^ii^	127.83 (16)	H22A—O22—H22B	99.00
O1—La1—O17^iii^	71.11 (14)	La1—O22—H22A	171.00
O1—La1—O5^i^	73.05 (13)	H27A—O27—H27B	104.00
O2—La1—O3	171.83 (16)	O1—C1—O2	119.2 (7)
O2—La1—O4	127.22 (14)	O1—C1—C2	118.0 (6)
O2—La1—O20	118.61 (16)	O2—C1—C2	122.8 (7)
O2—La1—O22	108.06 (15)	C3—C2—C7	119.8 (7)
O2—La1—O7^ii^	78.77 (16)	C1—C2—C3	120.7 (7)
O2—La1—O17^iii^	111.42 (13)	C1—C2—C7	119.4 (6)
O2—La1—O5^i^	68.20 (13)	C2—C3—C4	120.2 (9)
O3—La1—O4	48.67 (15)	C3—C4—C5	119.8 (9)
O3—La1—O20	66.90 (17)	C4—C5—C6	120.7 (9)
O3—La1—O22	67.90 (18)	C5—C6—C7	121.0 (9)
O3—La1—O7^ii^	93.09 (18)	C2—C7—C6	118.4 (7)
O3—La1—O17^iii^	74.93 (16)	C2—C7—C8	119.1 (6)
O3—La1—O5^i^	111.83 (16)	C6—C7—C8	122.4 (7)
O4—La1—O20	113.75 (15)	O17—C8—C7	116.9 (6)
O4—La1—O22	98.40 (15)	O18—C8—C7	118.5 (6)
O4—La1—O7^ii^	68.65 (15)	O17—C8—O18	124.6 (6)
O4—La1—O17^iii^	71.98 (14)	O4—C9—C12	117.6 (6)
O4—La1—O5^i^	67.52 (13)	O3—C9—O4	119.8 (7)
O20—La1—O22	65.81 (16)	O3—C9—C12	122.5 (7)
O7^ii^—La1—O20	135.44 (15)	O6—C10—C11	117.4 (6)
O17^iii^—La1—O20	79.05 (14)	O5—C10—C11	119.5 (6)
O5^i^—La1—O20	140.95 (14)	O5—C10—O6	123.1 (6)
O7^ii^—La1—O22	69.81 (16)	C10—C11—C16	119.5 (7)
O17^iii^—La1—O22	136.23 (15)	C12—C11—C16	119.6 (7)
O5^i^—La1—O22	152.50 (16)	C10—C11—C12	120.8 (6)
O7^ii^—La1—O17^iii^	135.85 (15)	C9—C12—C11	119.4 (7)
O5^i^—La1—O7^ii^	82.87 (15)	C9—C12—C13	120.9 (8)
O5^i^—La1—O17^iii^	63.96 (13)	C11—C12—C13	119.6 (8)
O1—La2—O17	71.82 (14)	C12—C13—C14	120.5 (11)
O1—La2—O19	84.69 (15)	C13—C14—C15	119.3 (10)
O1—La2—O4^iv^	164.86 (14)	C14—C15—C16	121.8 (10)
O1—La2—O5^iv^	123.63 (14)	C11—C16—C15	119.1 (9)
O1—La2—O8^v^	71.19 (14)	O7—C17—O8	123.7 (6)
O1—La2—O6^iii^	104.32 (14)	O8—C17—C18	119.0 (6)
O1—La2—O18^iii^	77.94 (14)	O7—C17—C18	117.1 (6)
O17—La2—O19	150.39 (15)	C17—C18—C23	122.3 (6)
O4^iv^—La2—O17	123.32 (14)	C17—C18—C19	117.9 (6)
O5^iv^—La2—O17	62.98 (13)	C19—C18—C23	119.2 (7)
O8^v^—La2—O17	114.56 (15)	C18—C19—C20	120.5 (7)
O6^iii^—La2—O17	70.92 (14)	C19—C20—C21	120.7 (9)
O17—La2—O18^iii^	87.01 (14)	C20—C21—C22	119.3 (9)
O4^iv^—La2—O19	80.80 (15)	C21—C22—C23	120.7 (8)
O5^iv^—La2—O19	121.13 (15)	C18—C23—C22	119.6 (7)
O8^v^—La2—O19	72.45 (17)	C18—C23—C24	120.4 (7)
O6^iii^—La2—O19	134.18 (15)	C22—C23—C24	119.9 (6)
O18^iii^—La2—O19	70.27 (15)	O19—C24—C23	116.4 (6)
O4^iv^—La2—O5^iv^	68.53 (14)	O20—C24—C23	118.2 (6)
O4^iv^—La2—O8^v^	100.10 (15)	O19—C24—O20	125.5 (7)
O4^iv^—La2—O6^iii^	83.05 (14)	C2—C3—H3	120.00
O4^iv^—La2—O18^iii^	100.99 (14)	C4—C3—H3	120.00
O5^iv^—La2—O8^v^	158.53 (14)	C5—C4—H4	120.00
O5^iv^—La2—O6^iii^	91.38 (14)	C3—C4—H4	120.00
O5^iv^—La2—O18^iii^	68.20 (14)	C4—C5—H5	120.00
O6^iii^—La2—O8^v^	68.66 (15)	C6—C5—H5	120.00
O8^v^—La2—O18^iii^	133.08 (15)	C7—C6—H6	120.00
O6^iii^—La2—O18^iii^	155.34 (14)	C5—C6—H6	119.00
La1—O1—La2	124.95 (16)	C12—C13—H13	120.00
La1—O1—C1	95.4 (4)	C14—C13—H13	120.00
La2—O1—C1	136.0 (4)	C15—C14—H14	120.00
La1—O2—C1	94.3 (5)	C13—C14—H14	120.00
La1—O3—C9	93.6 (5)	C14—C15—H15	119.00
La1—O4—C9	96.4 (4)	C16—C15—H15	119.00
La1—O4—La2^vi^	122.76 (16)	C15—C16—H16	120.00
La2^vi^—O4—C9	132.0 (4)	C11—C16—H16	120.00
La2^vi^—O5—C10	115.6 (4)	C18—C19—H19	120.00
La1^i^—O5—C10	127.7 (4)	C20—C19—H19	120.00
La1^i^—O5—La2^vi^	114.93 (16)	C21—C20—H20	120.00
La2^iii^—O6—C10	132.6 (4)	C19—C20—H20	120.00
La1^ii^—O7—C17	156.2 (5)	C20—C21—H21	120.00
La2^v^—O8—C17	110.5 (4)	C22—C21—H21	120.00
La2—O17—C8	112.8 (4)	C21—C22—H22	120.00
La1^iii^—O17—La2	118.14 (16)	C23—C22—H22	120.00
			
O2—La1—O1—La2	154.4 (3)	O5^iv^—La2—O19—C24	−74.0 (7)
O2—La1—O1—C1	−7.1 (3)	O8^v^—La2—O19—C24	124.4 (7)
O3—La1—O1—La2	−26.0 (3)	O6^iii^—La2—O19—C24	157.3 (6)
O3—La1—O1—C1	172.4 (4)	O18^iii^—La2—O19—C24	−26.6 (7)
O4—La1—O1—La2	−99.6 (2)	O17—La2—O4^iv^—La1^iv^	97.8 (2)
O4—La1—O1—C1	98.9 (4)	O17—La2—O4^iv^—C9^iv^	−122.9 (6)
O20—La1—O1—La2	18.1 (2)	O19—La2—O4^iv^—La1^iv^	−100.8 (2)
O20—La1—O1—C1	−143.5 (4)	O19—La2—O4^iv^—C9^iv^	38.4 (6)
O22—La1—O1—La2	71.7 (3)	O1—La2—O5^iv^—C10^iv^	−153.0 (4)
O22—La1—O1—C1	−89.8 (4)	O1—La2—O5^iv^—La1^iii^	40.9 (2)
O7^ii^—La1—O1—La2	163.59 (18)	O17—La2—O5^iv^—C10^iv^	166.4 (5)
O7^ii^—La1—O1—C1	2.1 (4)	O17—La2—O5^iv^—La1^iii^	0.34 (15)
O17^iii^—La1—O1—La2	−62.4 (2)	O19—La2—O5^iv^—C10^iv^	−47.3 (5)
O17^iii^—La1—O1—C1	136.0 (4)	O19—La2—O5^iv^—La1^iii^	146.65 (17)
O5^i^—La1—O1—La2	−130.1 (2)	O1—La2—O8^v^—C17^v^	−170.5 (5)
O5^i^—La1—O1—C1	68.4 (4)	O17—La2—O8^v^—C17^v^	−111.7 (4)
O1—La1—O2—C1	7.3 (3)	O19—La2—O8^v^—C17^v^	99.3 (5)
O4—La1—O2—C1	−113.4 (4)	O1—La2—O6^iii^—C10^iii^	−162.0 (5)
O20—La1—O2—C1	58.7 (4)	O17—La2—O6^iii^—C10^iii^	−97.4 (5)
O22—La1—O2—C1	130.4 (4)	O19—La2—O6^iii^—C10^iii^	101.4 (6)
O7^ii^—La1—O2—C1	−165.4 (4)	O1—La2—O18^iii^—C8^iii^	27.4 (6)
O17^iii^—La1—O2—C1	−30.3 (4)	O17—La2—O18^iii^—C8^iii^	−44.7 (6)
O5^i^—La1—O2—C1	−78.6 (4)	O19—La2—O18^iii^—C8^iii^	116.0 (7)
O1—La1—O3—C9	−107.5 (5)	La1—O1—C1—O2	13.1 (6)
O4—La1—O3—C9	7.1 (4)	La1—O1—C1—C2	−166.0 (5)
O20—La1—O3—C9	−156.2 (5)	La2—O1—C1—O2	−144.9 (5)
O22—La1—O3—C9	131.7 (5)	La2—O1—C1—C2	36.0 (9)
O7^ii^—La1—O3—C9	64.9 (5)	La1—O2—C1—O1	−12.9 (6)
O17^iii^—La1—O3—C9	−71.9 (5)	La1—O2—C1—C2	166.2 (6)
O5^i^—La1—O3—C9	−18.6 (5)	La1—O3—C9—O4	−12.6 (7)
O1—La1—O4—C9	115.6 (4)	La1—O3—C9—C12	163.6 (6)
O1—La1—O4—La2^vi^	−93.6 (2)	La1—O4—C9—O3	13.1 (7)
O2—La1—O4—C9	−177.8 (4)	La1—O4—C9—C12	−163.3 (6)
O2—La1—O4—La2^vi^	−27.0 (3)	La2^vi^—O4—C9—O3	−133.4 (6)
O3—La1—O4—C9	−6.9 (4)	La2^vi^—O4—C9—C12	50.2 (9)
O3—La1—O4—La2^vi^	143.9 (3)	La2^vi^—O5—C10—O6	−116.6 (6)
O20—La1—O4—C9	9.8 (4)	La2^vi^—O5—C10—C11	66.1 (7)
O20—La1—O4—La2^vi^	160.59 (18)	La1^i^—O5—C10—O6	47.4 (8)
O22—La1—O4—C9	−57.4 (4)	La1^i^—O5—C10—C11	−129.9 (5)
O22—La1—O4—La2^vi^	93.4 (2)	La2^iii^—O6—C10—O5	33.2 (9)
O7^ii^—La1—O4—C9	−121.8 (4)	La2^iii^—O6—C10—C11	−149.4 (5)
O7^ii^—La1—O4—La2^vi^	29.00 (19)	La1^ii^—O7—C17—O8	−113.2 (11)
O17^iii^—La1—O4—C9	78.6 (4)	La1^ii^—O7—C17—C18	61.0 (13)
O17^iii^—La1—O4—La2^vi^	−130.6 (2)	La2^v^—O8—C17—O7	29.7 (8)
O5^i^—La1—O4—C9	147.2 (4)	La2^v^—O8—C17—C18	−144.4 (5)
O5^i^—La1—O4—La2^vi^	−62.00 (19)	La2—O17—C8—O18	−110.4 (6)
O1—La1—O20—C24	19.1 (7)	La2—O17—C8—C7	70.1 (6)
O2—La1—O20—C24	−17.6 (8)	La1^iii^—O17—C8—O18	47.3 (8)
O3—La1—O20—C24	169.2 (8)	La1^iii^—O17—C8—C7	−132.3 (5)
O4—La1—O20—C24	155.6 (7)	La2^iii^—O18—C8—O17	27.6 (11)
O22—La1—O20—C24	−115.7 (8)	La2^iii^—O18—C8—C7	−152.9 (5)
O7^ii^—La1—O20—C24	−121.3 (7)	La2—O19—C24—O20	−34.9 (11)
O17^iii^—La1—O20—C24	91.0 (7)	La2—O19—C24—C23	144.8 (5)
O5^i^—La1—O20—C24	72.5 (8)	La1—O20—C24—O19	−17.0 (12)
O1—La1—O7^ii^—C17^ii^	26.2 (12)	La1—O20—C24—C23	163.3 (5)
O2—La1—O7^ii^—C17^ii^	33.3 (11)	O1—C1—C2—C3	−144.4 (7)
O3—La1—O7^ii^—C17^ii^	−147.4 (11)	O1—C1—C2—C7	33.0 (9)
O4—La1—O7^ii^—C17^ii^	−104.4 (11)	O2—C1—C2—C3	36.6 (10)
O20—La1—O7^ii^—C17^ii^	152.9 (10)	O2—C1—C2—C7	−146.1 (7)
O22—La1—O7^ii^—C17^ii^	147.5 (11)	C1—C2—C3—C4	175.6 (8)
O1—La1—O17^iii^—La2^iii^	−80.23 (19)	C7—C2—C3—C4	−1.7 (12)
O1—La1—O17^iii^—C8^iii^	76.4 (5)	C1—C2—C7—C6	−174.9 (6)
O2—La1—O17^iii^—La2^iii^	−50.9 (2)	C1—C2—C7—C8	7.9 (9)
O2—La1—O17^iii^—C8^iii^	105.7 (5)	C3—C2—C7—C6	2.4 (10)
O3—La1—O17^iii^—La2^iii^	123.7 (2)	C3—C2—C7—C8	−174.8 (7)
O3—La1—O17^iii^—C8^iii^	−79.7 (5)	C2—C3—C4—C5	0.1 (14)
O4—La1—O17^iii^—La2^iii^	72.88 (19)	C3—C4—C5—C6	0.8 (15)
O4—La1—O17^iii^—C8^iii^	−130.5 (5)	C4—C5—C6—C7	−0.1 (14)
O20—La1—O17^iii^—La2^iii^	−167.5 (2)	C5—C6—C7—C2	−1.5 (11)
O20—La1—O17^iii^—C8^iii^	−10.9 (5)	C5—C6—C7—C8	175.6 (8)
O22—La1—O17^iii^—La2^iii^	156.13 (19)	C2—C7—C8—O17	−110.5 (7)
O22—La1—O17^iii^—C8^iii^	−47.3 (6)	C2—C7—C8—O18	69.9 (8)
O1—La1—O5^i^—La2^iii^	77.17 (18)	C6—C7—C8—O17	72.4 (9)
O1—La1—O5^i^—C10^i^	−118.7 (5)	C6—C7—C8—O18	−107.2 (8)
O2—La1—O5^i^—La2^iii^	129.6 (2)	O3—C9—C12—C11	−145.8 (8)
O2—La1—O5^i^—C10^i^	−66.3 (5)	O3—C9—C12—C13	32.4 (12)
O3—La1—O5^i^—La2^iii^	−59.2 (2)	O4—C9—C12—C11	30.5 (10)
O3—La1—O5^i^—C10^i^	104.9 (5)	O4—C9—C12—C13	−151.3 (8)
O4—La1—O5^i^—La2^iii^	−79.85 (18)	O5—C10—C11—C12	−99.6 (8)
O4—La1—O5^i^—C10^i^	84.3 (5)	O5—C10—C11—C16	85.3 (9)
O20—La1—O5^i^—La2^iii^	20.6 (3)	O6—C10—C11—C12	82.9 (9)
O20—La1—O5^i^—C10^i^	−175.3 (5)	O6—C10—C11—C16	−92.3 (8)
O22—La1—O5^i^—La2^iii^	−143.0 (3)	C10—C11—C12—C9	1.6 (11)
O22—La1—O5^i^—C10^i^	21.1 (7)	C10—C11—C12—C13	−176.6 (7)
O17—La2—O1—La1	127.4 (2)	C16—C11—C12—C9	176.8 (7)
O17—La2—O1—C1	−79.6 (6)	C16—C11—C12—C13	−1.5 (12)
O19—La2—O1—La1	−34.3 (2)	C10—C11—C16—C15	178.2 (8)
O19—La2—O1—C1	118.7 (6)	C12—C11—C16—C15	3.0 (12)
O5^iv^—La2—O1—La1	89.9 (2)	C9—C12—C13—C14	−177.9 (9)
O5^iv^—La2—O1—C1	−117.2 (5)	C11—C12—C13—C14	0.3 (13)
O8^v^—La2—O1—La1	−107.6 (2)	C12—C13—C14—C15	−0.6 (16)
O8^v^—La2—O1—C1	45.4 (5)	C13—C14—C15—C16	2.3 (16)
O6^iii^—La2—O1—La1	−168.64 (18)	C14—C15—C16—C11	−3.4 (15)
O6^iii^—La2—O1—C1	−15.7 (6)	O7—C17—C18—C19	−128.0 (7)
O18^iii^—La2—O1—La1	36.59 (19)	O7—C17—C18—C23	43.1 (9)
O18^iii^—La2—O1—C1	−170.4 (6)	O8—C17—C18—C19	46.5 (9)
O1—La2—O17—C8	13.8 (4)	O8—C17—C18—C23	−142.4 (6)
O1—La2—O17—La1^iii^	−145.6 (2)	C17—C18—C19—C20	169.6 (7)
O19—La2—O17—C8	53.0 (5)	C23—C18—C19—C20	−1.8 (11)
O19—La2—O17—La1^iii^	−106.4 (3)	C17—C18—C23—C22	−169.5 (7)
O4^iv^—La2—O17—C8	−166.7 (4)	C17—C18—C23—C24	12.1 (10)
O4^iv^—La2—O17—La1^iii^	33.9 (2)	C19—C18—C23—C22	1.5 (10)
O5^iv^—La2—O17—C8	159.1 (5)	C19—C18—C23—C24	−176.9 (6)
O5^iv^—La2—O17—La1^iii^	−0.36 (16)	C18—C19—C20—C21	1.4 (13)
O8^v^—La2—O17—C8	−44.7 (4)	C19—C20—C21—C22	−0.6 (14)
O8^v^—La2—O17—La1^iii^	155.92 (17)	C20—C21—C22—C23	0.2 (14)
O6^iii^—La2—O17—C8	−99.1 (4)	C21—C22—C23—C18	−0.7 (12)
O6^iii^—La2—O17—La1^iii^	101.5 (2)	C21—C22—C23—C24	177.7 (8)
O18^iii^—La2—O17—C8	92.1 (4)	C18—C23—C24—O19	46.1 (9)
O18^iii^—La2—O17—La1^iii^	−67.35 (19)	C18—C23—C24—O20	−134.2 (7)
O1—La2—O19—C24	52.5 (7)	C22—C23—C24—O19	−132.3 (7)
O17—La2—O19—C24	15.4 (9)	C22—C23—C24—O20	47.4 (10)
O4^iv^—La2—O19—C24	−131.9 (7)		

Symmetry codes: (i) −*x*+1, −*y*+1, −*z*+1; (ii) −*x*, −*y*, −*z*+1; (iii) −*x*, −*y*+1, −*z*+1; (iv) *x*−1, *y*, *z*; (v) −*x*−1, −*y*, −*z*+1; (vi) *x*+1, *y*, *z*.

### Hydrogen-bond geometry (Å, °)

**Table:**

Cg is the centroid of the C2–C7 ring.

**Table:**

*D*—H···*A*	*D*—H	H···*A*	*D*···*A*	*D*—H···*A*
O22—H22A···O1^ii^	0.90	2.20	3.001 (6)	148.
O22—H22B···O20^ii^	0.88	2.15	3.009 (8)	164.
O27—H27A···O2^vii^	0.91	2.38	3.17 (2)	146.00
O27—H27B···O3^i^	0.88	1.86	2.69 (2)	158.00
C3—H3···O27^viii^	0.93	2.15	2.96 (2)	145.
C16—H16···O2^i^	0.93	2.58	3.331 (10)	138.
C19—H19···Cg^v^	0.93	2.98	3.902 (9)	169.

Symmetry codes: (ii) −*x*, −*y*, −*z*+1; (vii) *x*+1, *y*+1, *z*; (i) −*x*+1, −*y*+1, −*z*+1; (viii) *x*−1, *y*−1, *z*; (v) −*x*−1, −*y*, −*z*+1.

## References

[bb1] Nonius (2000). *COLLECT* Nonius BV, Delft, The Netherlands.

[bb2] Otwinowski, Z. & Minor, W. (1997). *Methods in Enzymology*, Vol. 276, *Macromolecular Crystallography*, Part A, edited by C. W. Carter Jr & R. M. Sweet, pp. 307–326. New York: Academic Press.

[bb3] Sheldrick, G. M. (2008). *Acta Cryst.* A**64**, 112–122. 10.1107/S0108767307043930 18156677

[bb4] Spek, A. L. (2009). *Acta Cryst.* D**65**, 148–155. 10.1107/S090744490804362X PMC2631630 19171970

[bb5] Wang, G.-M., Xue, S.-Y., Li, H. & Liu, H.-L. (2009). *Acta Cryst.* C**65**, m469–m471. 10.1107/S0108270109043856 19966429

